# Forensic analysis of beverage stains using hyperspectral imaging

**DOI:** 10.1038/s41598-021-85737-x

**Published:** 2021-03-22

**Authors:** Binu Melit Devassy, Sony George

**Affiliations:** Department of Computer Science, Norwegian University of Science and Technology (NTNU), 2802 Gjøvik, Norway

**Keywords:** Imaging and sensing, Optical spectroscopy, Imaging techniques, Near-infrared spectroscopy, Characterization and analytical techniques

## Abstract

Documentation and analysis of crime scene evidences are of great importance in any forensic investigation. In this paper, we present the potential of hyperspectral imaging (HSI) to detect and analyze the beverage stains on a paper towel. To detect the presence and predict the age of the commonly used drinks in a crime scene, we leveraged the additional information present in the HSI data. We used 12 different beverages and four types of paper hand towel to create the sample stains in the current study. A support vector machine (SVM) is used to achieve the classification, and a convolutional auto-encoder is used to achieve HSI data dimensionality reduction, which helps in easy perception, process, and visualization of the data. The SVM classification model was re-established for a lighter and quicker classification model on the basis of the reduced dimension. We employed volume-gradient-based band selection for the identification of relevant spectral bands in the HSI data. Spectral data recorded at different time intervals up to 72 h is analyzed to trace the spectral changes. The results show the efficacy of the HSI techniques for rapid, non-contact, and non-invasive analysis of beverage stains.

## Introduction

For a crime scene investigator, it is crucial to find the presence of all micro–macro scale physical, chemical, and biological evidences in the scene. These evidences assist in describing what incidences led to the criminal activity. In many cases, criminal behavior occurred under the influence of alcoholic beverages or used the drinks as a means of poisoning or sedating victims to facilitate sexual abuse or robbery^1–3^. In evaluating the crime scene and modeling the suspect or victim´s actions, the identification and analysis of widely used drinks such as coffee, beer, and cocktails play a critical role. The detection of human body fluids such as blood, sweat, urine, saliva, and semen are previously studied by various researchers^4–6^, however, the presence of beverage stains are seldom examined^7^. In addition, examination of a paper towel collected from the crime scene will provide evidence relevant to the crime ^8,9^ such as information about fingerprints, the tools used for crime, body fluid samples, and the beverages used. Aging analysis of stains^10^ also plays a vital role in crime analysis to predict the appropriate timelines of a crime. Aging of stains from human body fluids are studied several times^11–13^ and some of them are capable of predicting aging up to a couple of years^14^, however the application of scientific imaging techniques for understanding the aging of beverage stain is not yet analyzed.

**Figure 1:**
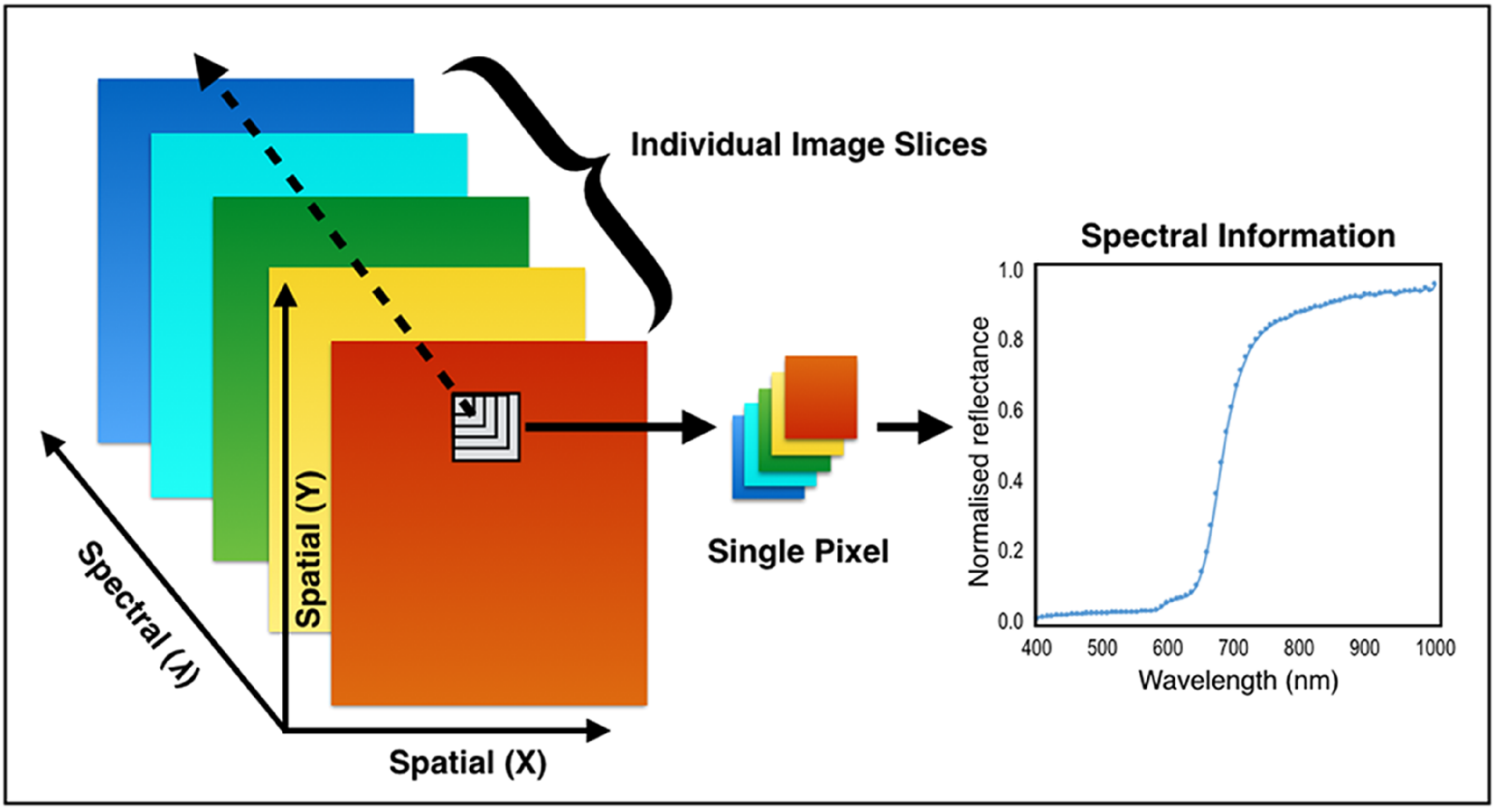
Hyperspectral image data representation^25^.

Existing methods for detecting the presence of beverage stains are mainly destructive chemical methods^7^ which require contact with the samples, however, forensic analysts prefer to have contactless and non-destructive techniques that preserve the evidence even after the analysis. Imaging techniques offer these desired qualities^15–17^, however lack the ability to record accurate appearance of the scene. Hence, in this work we investigated the potential of hyperspectral imaging (HSI) to detect and analyze the beverages from the paper towel. The HSI blends ordinary imaging with spectroscopy, which can simultaneously collect both spectral and spatial data^18^, HSI can provide much more details about the material under investigation with the aid of additional information along the spectral axes compared to conventional imaging techniques. HSI was initially developed for use in satellite imaging^19^, but later evolved as an important tool in many fields such as food processing^20^, agriculture^21^, cultural heritage^22^, medical imaging^23^ and forensics^24^. HSI data consists of different band images as layers and sometimes called as an HSI data cube having spatial information along X and Y-axes and spectral information along Z-axis as in Fig. 1. Each point on the HSI data cube characterizes the material property by having a spectrum that can be regarded as a specific material signature and can be used to identify the material. As mentioned, HSI already used in many forensic applications such as bloodstain analysis^10^, ink and paper analysis^25,26^, fingerprint detection^27^, and face detection^28^. However, the potential of HSI imaging has not yet been extended to forensic analysis of beverage stains, and through this work, we are trying to exploit the potential of HSI in this direction.

The HSI capture data over a broad spectral region, and this leads to challenges in handling the data for visualization, storage, and more computating power is needed for analysis. Dimensionality reduction is therefore considered an essential preprocessing phase in HSI research, which will also allow the classifier to construct a detailed model at minimum computational expenditure. Visualization is another important outcome of dimensionality reduction, it is expected in many forensic cases that the number of stains present is unclear, visualization of the data gives some clue about this, it also allows the investigator to schedule the appropriate processing pipeline. Principal component analysis (PCA)^29^ and independent component analysis (ICA)^30^ are the most widely used dimensionality reduction techniques, but because of their simple linearity assumption, they are more suited for a linear data set^31^. In this work, we used convolutional auto-encoder (CAE)^32^ as a dimensionality reduction tool because of its ability to provide a more efficient and nonlinear generalization of the input data than PCA^33^. To achieve an efficient dimensionality reduction, the CAE learns its parameters during the training process and then the convolutional encoder (CE)^34^ will be separated from CAE and used for dimensionality reduction.

The main objective of this research is to investigate the HSI's capability for the detection and classification of beverage stains at different periods, with the aid of support vector machines (SVM) and CAE. In addition, we also evaluate the spectral changes that occur over 3 days for each drinks. The volume-gradient-based band selection (VGBS)^35^ algorithm is used to detect the significant bands in the HSI data acquired, VGBS is suitable for unsupervised band selection. The remaining part of this paper is organized as follows: in second section, “Materials and methods” used for the study are listed, “Results and discussion” are described in next section, and in last section, “Conclusions” of this study are presented.

## Materials and methods

### Materials

**Figure 2:**
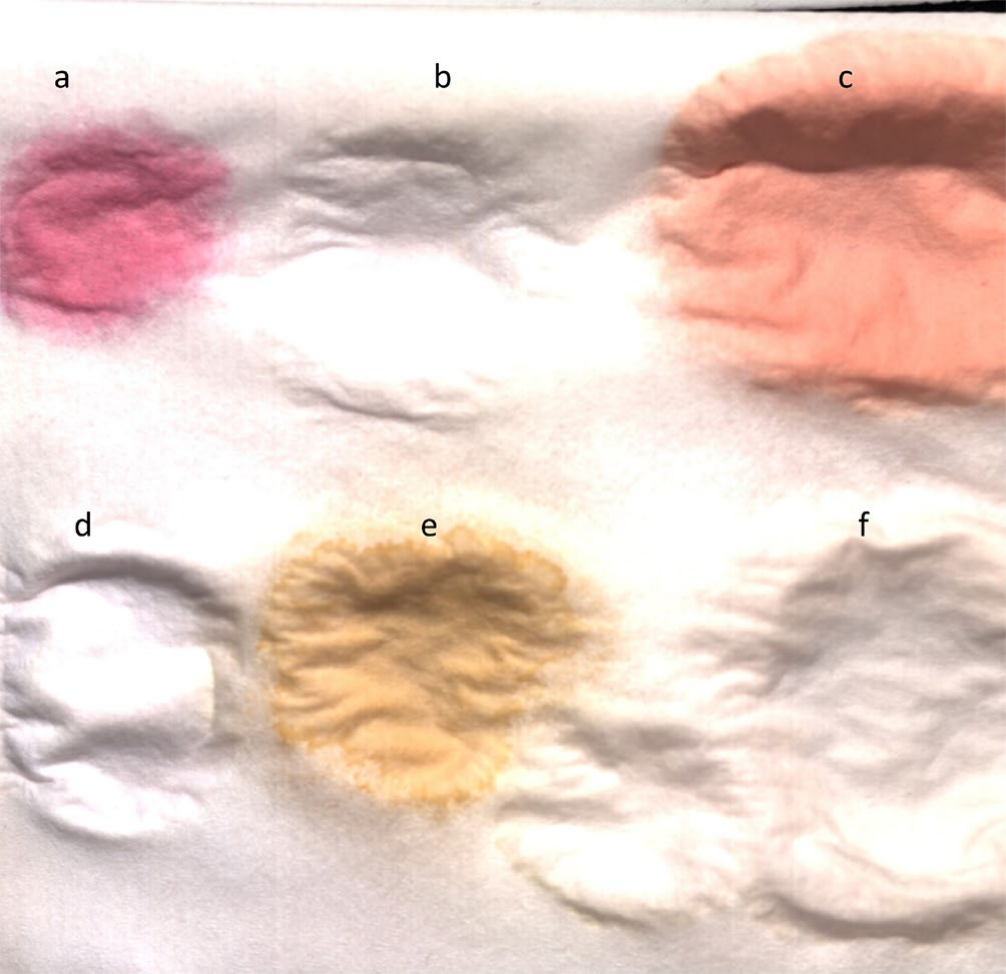
Paper1, wine samples in the paper are marked by a, b, & c and d, e and f are corresponding to Martini, coffee, and beer.

We have prepared the samples for the study in the lab, which allows to understand the contents better and later evaluating the algorithms effectively. Four different types of paper towels were used as a substrate for creating the sample stains. Six beverage samples were used in the first paper (paper1) and twelve beverage samples in the other three paper towel types (paper2, paper3, and paper4). The paper1 was used to study the beverage stains spectral changes over time and others were used for classification. Three samples from each paper type (paper2, paper3 and paper4) were collected in order to perform the classification tasks. The beverages used on the paper1 are white wine (Weingut Robert Weil), beer (Aass Fatøl), port wine (Velhotes), Martini (Bianco Vermouth), coffee (Green Forest, Arvid Nordquist), and red wine (Altano). For classification task, we used the same beverages except white wine and beer (Aass Fatøl) along with tea (Yellow Label, Lipton), Fanta, Coca-Cola, beer1 (Heineken), beer2 (Corona Light), orange juice (freshly squeezed), apple juice (Balholm) and dry Jin (Bombay Sapphire). As shown in Fig. 2, samples from all these selected drinks were poured onto the paper towel. The HSI of the samples were acquired at 0, 15, 30 min and 1, 3, 24, 48 and 72 h. The paper towel with stain samples was kept at room temperature during this period. To replicate the real scenario, we have not followed any procedures to avoid the surface shrinking of the paper, which happens due to the drying of the stains.

### Hyperspectral image acquisition

**Figure 3:**
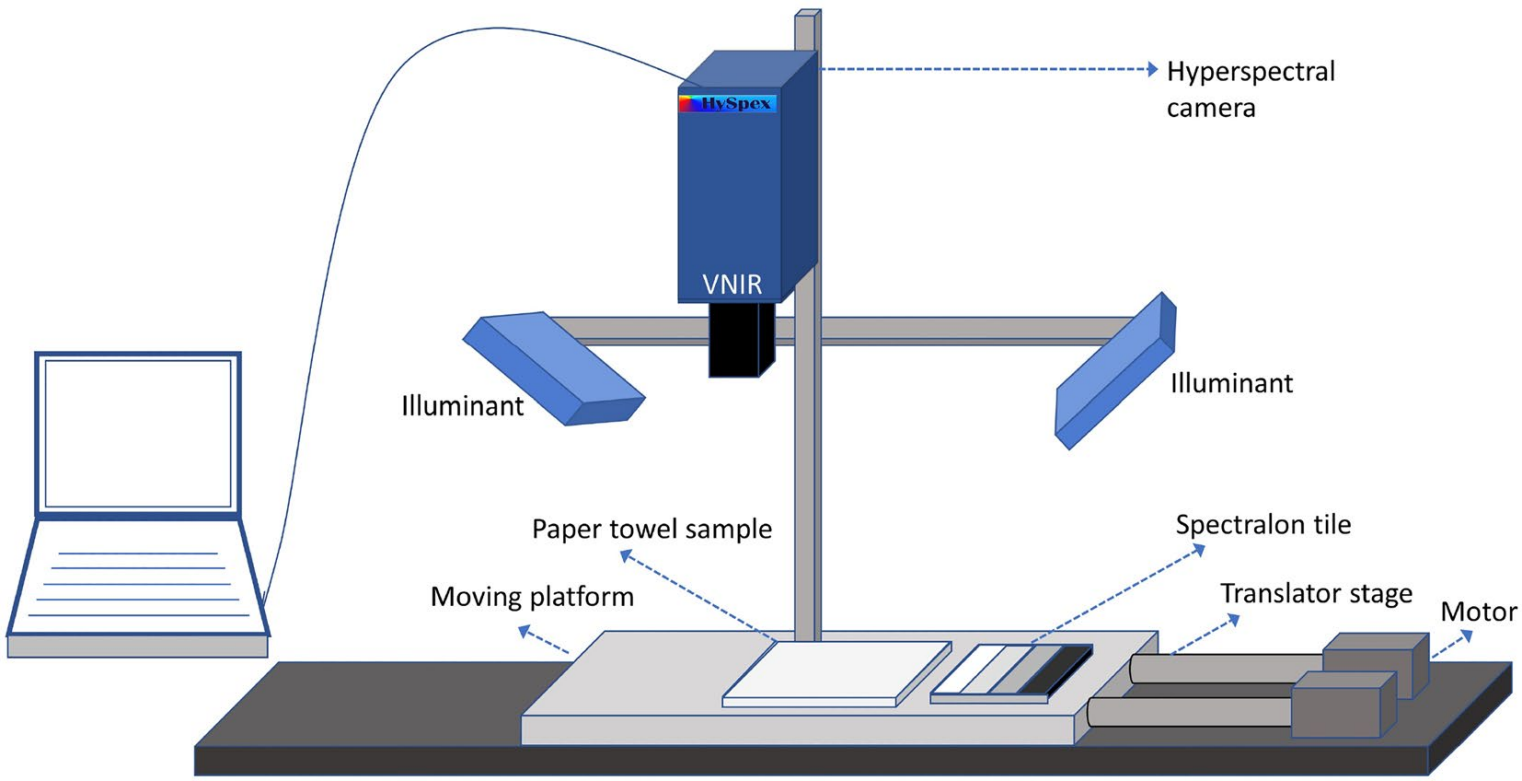
Hyperspectral image acquisition setup.

A push broom hyperspectral camera HySpex VNIR-1800^36^, developed by Norsk Elektro Optikk AS, is used for the HSI acquisition. This camera has sensitivity over a spectral range of 400–1000 nm with a spectral resolution of 3.18 nm, which results in a spectral dimension of 186 (bands). The imaging system records 1800-pixels across the field of 10 cm in the configuration we used for the present study. The sample paper towel was placed at a distance of 30 cm from the camera on a translator stage as, in Fig. 3. This moving stage was synchronized with the integration time of the camera. The setup consists of two halogen light sources with 45°:0° geometry with respect to the camera to illuminate the sample. A contrast multi-step target^37^ of known reflectance was placed next to the paper towel and this was used later for estimating the normalized reflectance of the sample. We used a polarizer in front of the lens while acquiring samples of the paper types except for paper1.

### Convolutional auto-encoder

**Figure 4:**
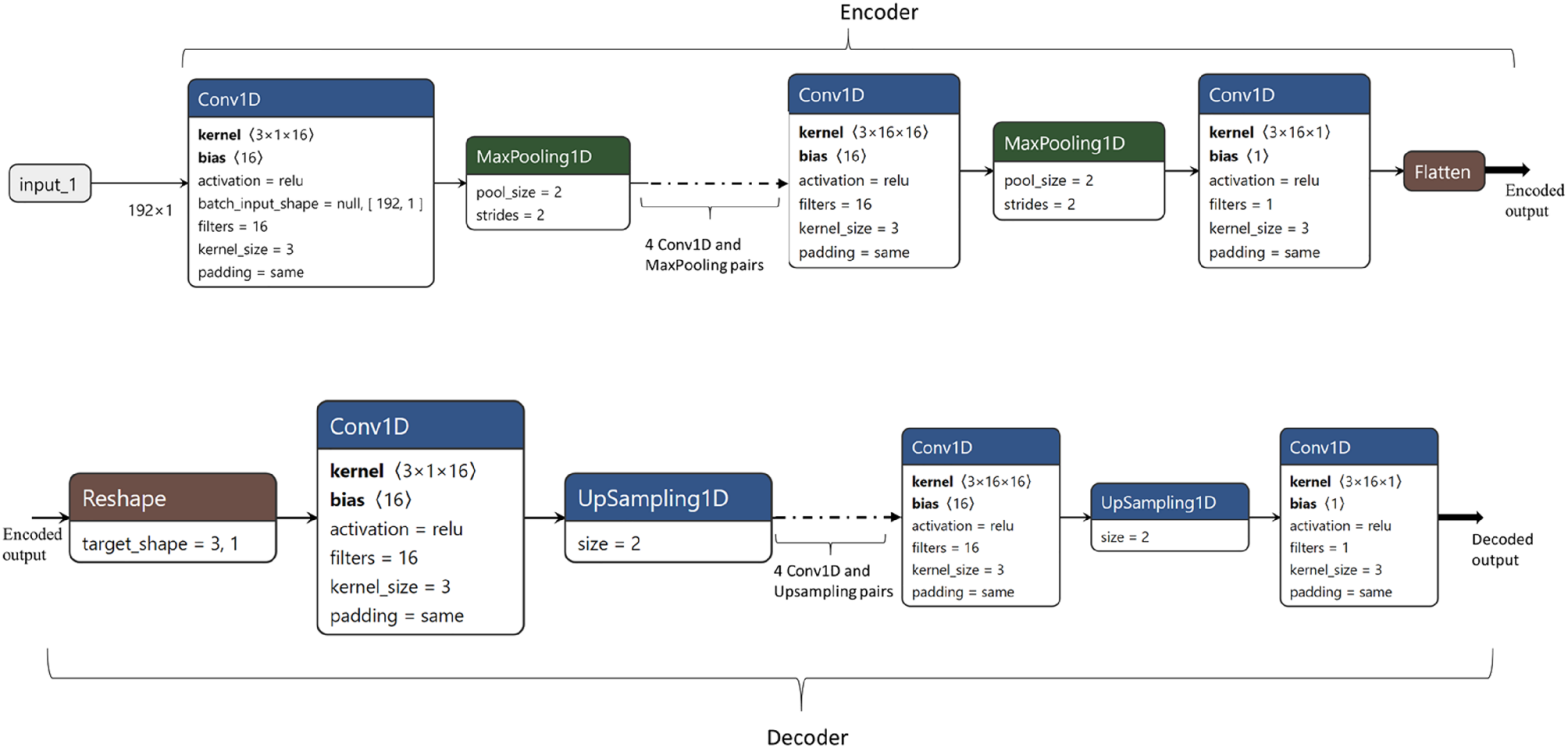
Convolutional auto-encoder (CAE) architecture, generated using Netron^39^.

An auto-encoders (AE) is an algorithm for extracting features based on an unsupervised neural network, which learns the best parameters possible for rebuilding its output as close as possible to its input^33^. AEs are extensively used in dimensionality reduction and feature extraction problems^38^, however a CAE is a special type of auto-encoder which employs convolutional neural networks (CNN) for achieving this. A CAE consists of an encoder and a decoder part, usually the decoder design follows a mirror image of encoder with down sampling replaced with up-sampling. The input will be passed to the encoder which compress the data into a smaller dimension and then the decoder learns to recreates the input from the encoded signal by adjusting parameters. The error (loss) will be calculated as the difference between decoded output and the input signal, here we used mean squared error (MSE) as a loss function. Here we considered that each spectrum as a vector having a dimension equal to the number of bands (186), the encoder compresses the vector to three-dimensional (3D) space and the decoder try to reconstruct the original vector from the encoded 3D data. During training the encoder and decoder parameters will learn to obtain a minimal loss. The detailed architecture of our CAE design is shown in Fig. 4, encoder part is designed in a way that the input data will be compressed to half at each level, a level consists of a pair of a 1-dimensional CNN with ReLU (rectified linear unit) as activation and a Maxpooling layer. To achieve this size reduction, the input vector is augmented to 192 dimensions by duplicating last feature, which originally has 186 dimensions. Even though CAE is designed to get a 3D encoder output, by removing a few pairs of Conv1D and sampling layers from both the encoder and the decoder, it is also possible to get higher output dimensions from the encoder. Here, we used six-dimension (6D) for evaluating classification capabilities of encoded data using SVM, the input spectra was compressed to 6D by removing a pair of Conv1D and Maxpooling from encoder side and a pair of Conv1D and Upsampling from decoder side.

### Data analysis

The processing starts with camera software, which applies sensor and radiometric corrections along with dark current reduction. For each instance, the HSI camera provides an HSI data cube with a resolution of 1800 × 11,144 × 186 (width × length × bands) pixels and a dynamic range of 16-bit unsigned integers. The HSI data was converted to normalized reflectance (floating point, 32 bit) values between 0 and 100% during the normalization process, representing the spectral response at each pixel which is calculated with the aid of the known reflectance values obtained from the multilevel reference target present in the scene. As seen in the Eq. (1), the spectral reflectance value can be computed as the ratio of reflected to incident light.

1 $$R\left(x,y,\lambda \right)=\frac{{L}_{r}\left(x,y,\lambda \right)}{{L}_{i}(x,y,\lambda )}$$

Here R(x, y, λ) is the reflectance for wavelength λ at location x, y, the Li and Lr are the incident and reflected lights for wavelength λ at x, y. Equation (1) can be re-arranged to determine the incident light as below.

2 $${L}_{i}\left(x,y,\lambda \right)=\frac{{L}_{r}\left(x,y,\lambda \right)}{R\left(x,y,\lambda \right)}$$

Incident light across the field of view will be calculated using the known reflectance of the reference target and reflected light intensities obtained from HSI camera by applying Eq. (2). Since the camera is a line scanner, we can reuse the incident light to calculate reflectance of the entire HSI image using Eq. (1). The next step is to select the center points of each type of beverage stain in the paper towel, which later used to extract the spectrum. A square-shaped region of interest (ROI) having a side length of 25 pixels around the center point of each stain is used. The size is selected based on the maximum possible ROI area for all stain present in the sample. For each type of beverage, the ROI will be used for extracting spectra, 80%of each stain spectrum has been used for training and 20% has been used to evaluate the proposed method. Accuracy is used as the parameter for assessing the classification capacity of the system, which can be defined as the fraction between the results predicted correctly (true positive + true negative) and the sum of all predictions.

**Figure 5:**
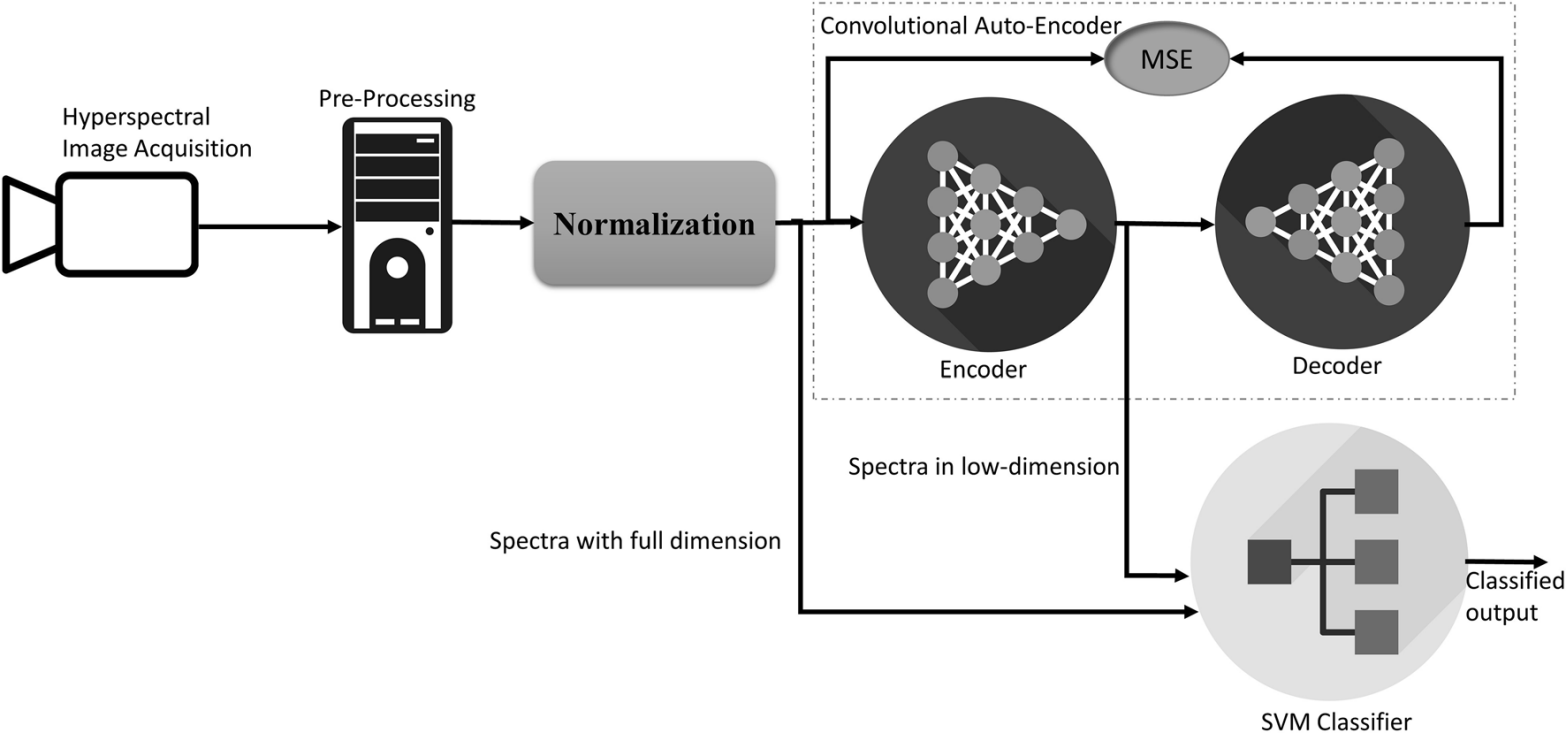
High level architecture of processing pipeline, the classifier accepts either one of the input based on settings.

Figure 5 describes the data flow pipeline, the normalized spectral data from the samples were used to train the CAE with MSE loss function, the CAE trained to minimize the error. After the learning phase, the encoder part is detached from CAE and used for encoding the input spectra into a lower dimension. In order to train the SVM to detect beverages, 80% spectra from each sample at a particular time duration will be fed to SVM and the remaining 20% (not used for training) will be used to evaluate the accuracy. To make a lighter and faster classification, the output from the encoder is used to train the SVM similar to the previous full spectral band training and testing. To classify the stains based on age, spectrum of each beverage at different time duration is extracted and followed similar strategy for training and testing that of identification of beverage stain.

A number of optimal wavelengths were chosen to create multispectral imaging systems in order to eliminate redundant information for the realization of HSI in future online inspections^40^. To remove the redundant bands, we selected volume-gradient-based band selection (VGBS), which is an unsupervised method attempts to successively remove the most redundant bands^35^. We selected this algorithm as a candidate because of its minimal computational complexity and popularity in band selection^41–43^ applications. In regards to a HSI, the VGBS uses a “subtle relationship between the volume of a subsimplex (or a subparallelotope) and the volume gradient of a simplex (or a parallelotope)”^35^, which makes the VGBS method extremely time efficient. In particular, while the VGBS method aims to find the band set with the largest volume, it does not need to measure any volume or even the distance spanned by the other bands between one band and the hyperplane. Instead, VGBS removes the most redundant bands, only based on the volume gradient.

## Results and discussion

### Spectral variation over time

**Figure 6:**
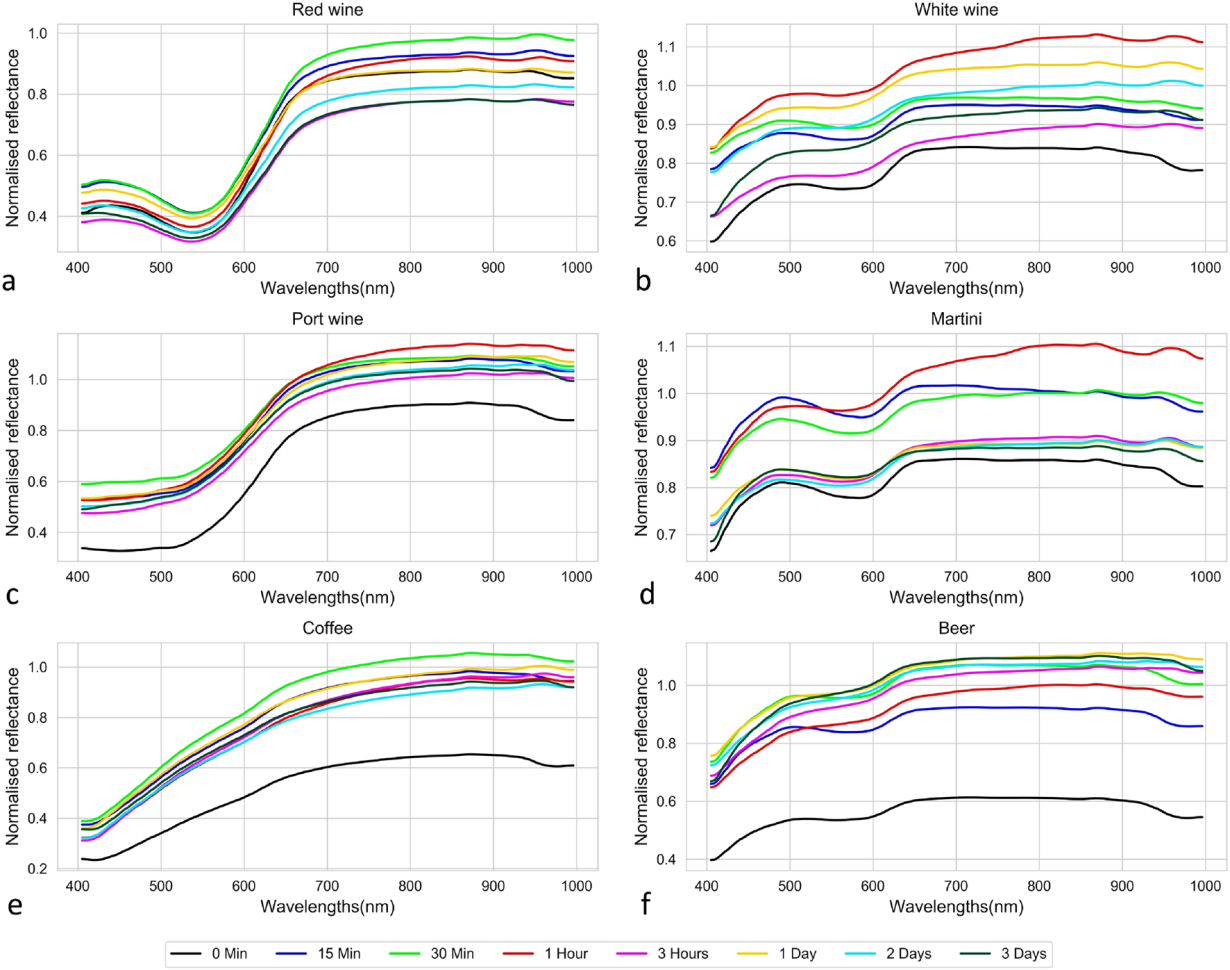
Spectral reflectance changes of beverage stains over time (paper1).

Mean spectral reflectance curves of the six beverages were determined from the proposed ROI over 3 days from paper1, and shown in Fig. 6, they have different patterns of reflectance spectrum variance as a function of time. The spectral variation of the red wine sample over 72 h (3 days) is represented in Fig. 6a, with less variation between the mean spectra in the visible region (400–700 nm) relative to that in the near infrared (NIR) region over the observation period. They have a low reflectance values up to 550 nm, and the reflectance curve increases steadily up to 700 nm (approximately) and retains a reasonably high reflectance values up to 1000 nm. It was also noted that the spectral reflectance variation over time is unpredictable. However, while their reflectance values are different, all the spectrum has nearly the same spectral shape. The spectrum of white wine is shown in Fig. 6b, it has more significant changes over time duration relative to red wine. It has also been noted that these changes do not follow any pattern over time. Another important finding is that spectral data has saturation at certain wavelengths, where the reflectance value moved beyond the upper limit of the normalized reflectance (1.0). This shows the presence of specular reflection that is obtained over time from the liquid residue and the irregular bumps that form over the paper surface.

The spectral changes of port wine is seen in Fig. 6c, where the NIR response is almost identical to that of red wine with saturated pixels in the NIR range. But in the visible region, spectrum from both exhibit a significant change. Figure 6d illustrates the Martini spectrum that follows a similar NIR features of white wine, both of which have an almost similar spectral shape in the NIR region. However, both of them possess distinct spectral features in visible range. Figure 6e is the standard coffee spectrum^44^, and aside from the initial spectrum, the variations over time are marginal for coffee spectrum. Finally, Fig. 6f possesses the spectra of beer, like coffee except for initial spectrum all others possess nearly identical spectra. In general, we found that changes in the spectral signature of the samples occurred over time, but for all the six beverages used in this analysis, no general patterns of changes for spectrum over time were noticed. After a visual review, we found that the spectral changes occurring in the last three timings appear marginal for the last four drinks, they are port wine, martini, coffee, and beer. The mean spectra of six beverage stains are significantly different from each other especially in the visible region and towards the end of the NIR region.

### Dimensionality reduction using encoder

**Figure 7:**
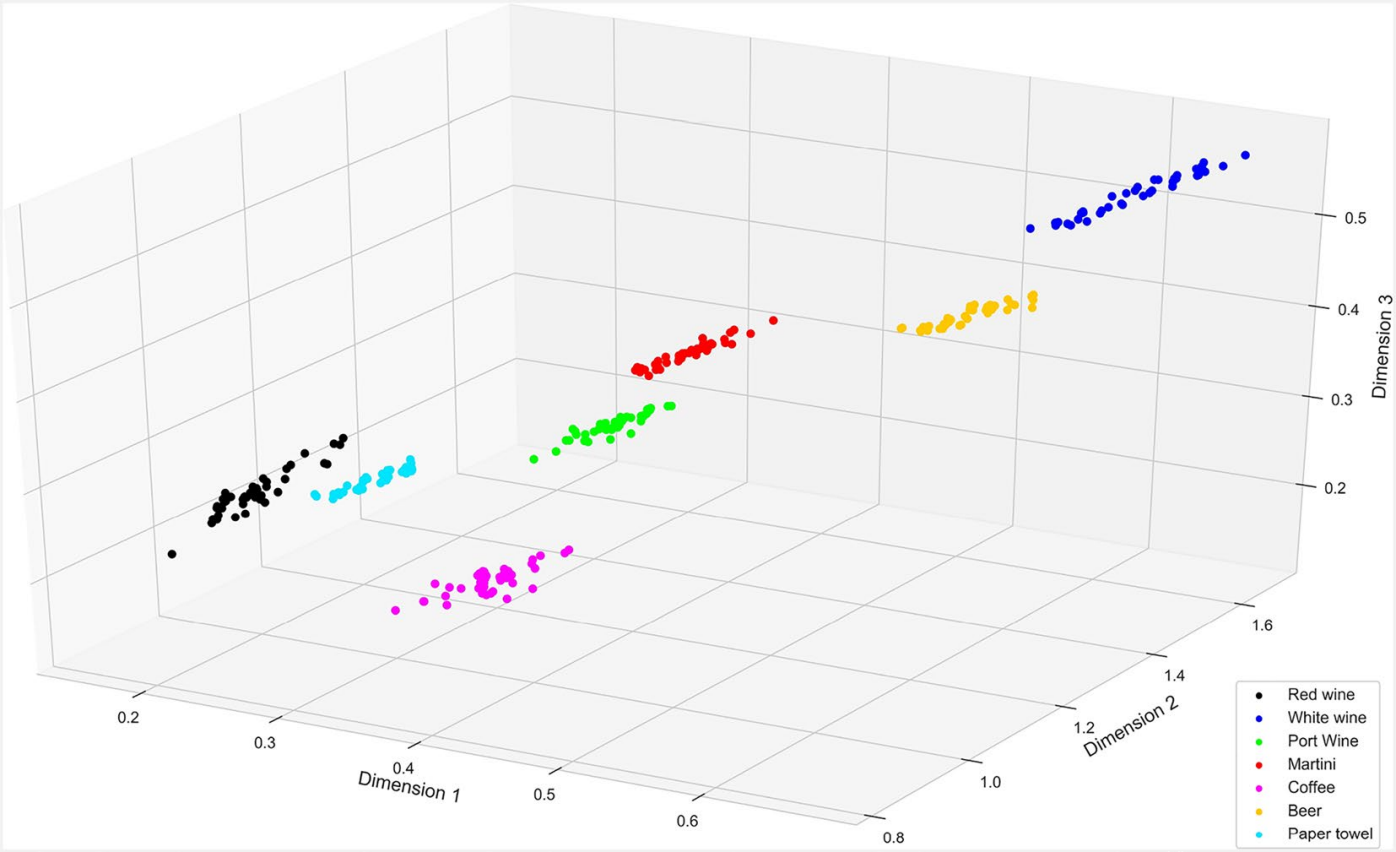
Encoder output for paper1 (ROI 20)—spectra in lower dimensions.

The CAE model was implemented, trained, and tested on the HSI data set of the beverage stains. The proposed CAE architecture was implemented using Keras^45^ in Python. The encoder, which is disconnected from the CAE, is used after training to reduce the dimensionality of spectral data. From the original dimension of 186, the encoder will reduce the input spectra to a lower three-dimensional space, which can then be visualized using a three-dimensional plot, as shown in Fig. 7. This form of visualization will give the investigator some clues about the possible number of materials present in the study sample. In this particular case, from Fig. 7 we can deduce the number of materials as seven, including paper towel, this information is useful in many cases where we do not have any prior knowledge about the number of samples present in the scene. Therefore, each cluster is well differentiated from other clusters a classifier can effortlessly separate these materials. Also, the clustering quality of the encoder output was measured using the Silhouette Index (SI)^46^ and normalized mutual information (NMI)^47^ and obtained 0.55 and 0.81 for SI and NMI, respectively. Both indexes define a good clustering, if the measured clustering index value is close to one. The values obtained here are moderately suitable for both indexes and which indicates the clustering obtained in this particular case is acceptable.

### Classification results

**Figure 8:**
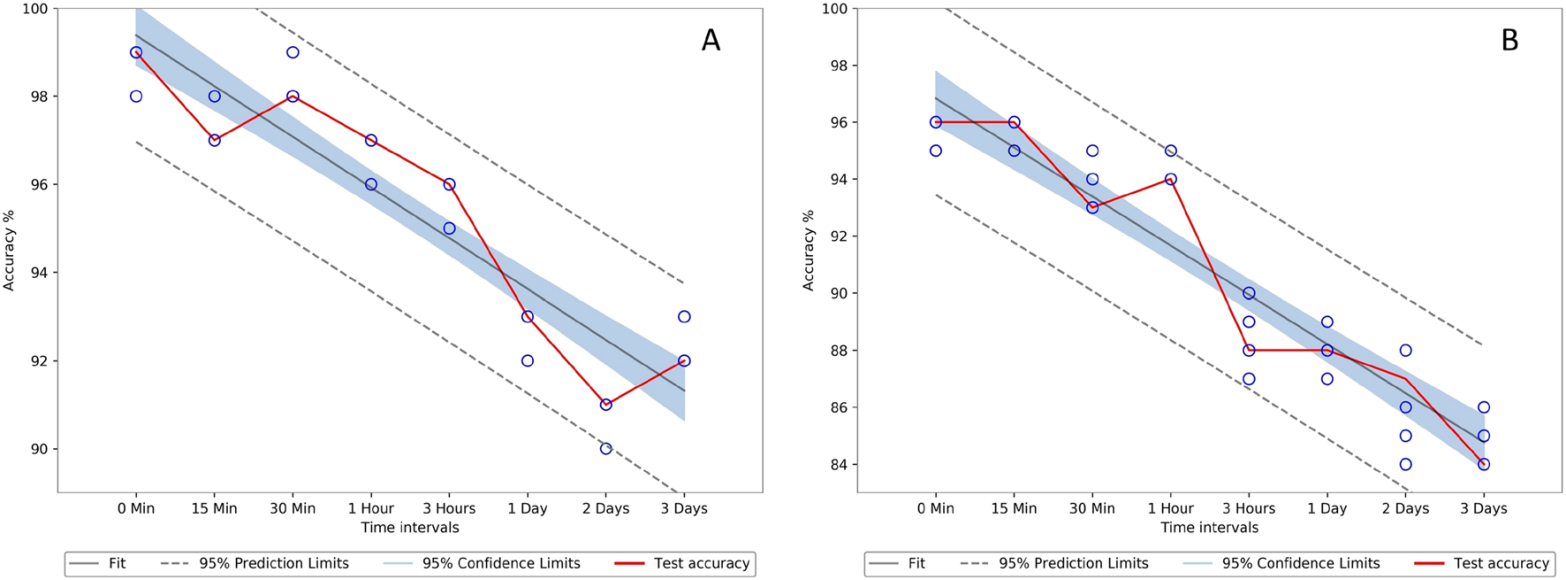
Paper3 confidence interval (CI) plot for beverage classification, (**A**) represents CI for SVM only and (**B**) represents CI for SVM with encoder.

Three samples collected from paper2, paper3 and paper4 were used for calculating classification accuracies. The average spectrum of all 12 beverage stains obtained is included in Appendix (Fig. 11). The SVM classifier with polynomial kernel was used to achieve the classification goals. The first objective was to classify the beverage stain, and the second was to detect the stains aging. We have used fivefold cross-validation to calculate the average accuracy and computation time on training data (80% of total data) and used the test data to calculate the test accuracy. For the first case, the SVM model was applied using full spectral data, considering the reflectance data as X variables and the stain type (beverage type) as Y variables. As shown in Table 1, the SVM models obtained convincing results with the overall classification accuracy of 94.40% on training data and 93.30% on test data sets. However, the calculation time will be influenced by the high dimensionality of the input spectrum (186), and the redundant characteristics can affect the robustness of the classification at a later level. Therefore, we attempted to detect beverage stains with the low dimensional data (used six dimension) obtained from the encoder output and obtained an average training accuracy of 89.50% and a test accuracy of 89.10%, which is extremely useful in forensic applications^48–50^. These values are much closer to that with full spectrum accuracy with approximately 5 times improvement in training time and 62 times lower memory usage. Table 1 provides detailed information on the average training time taken, calculated on a computer with an Intel Core i7 8650U CPU and 16 GB of RAM. The confidence interval (CI) plots give more detailed information about the variation of accuracies between different folds in k-fold validation. The Fig. 8 depicts the CI of the paper3 and the CI of other papers are given in Appendix as Figs. 12 and 13. The Table 2 gives the Analysis of variance (ANOVA) of the accuracies obtained over k-fold validation, all the p-values are greater than 0.05 implies that there is no significant differences between the accuracies.

**Table 1:** Accuracies (average) for detecting beverage stains and processing time on the training data.

	0 min	15 min	30 min	1 h	3 h	1 day	2 days	3 days	Average
**SVM with encoder**									
Acc %	93.67	92.40	90.67	91.80	90.33	87.67	82.80	86.67	89.50
Time (s)	1.2	1.3	0.9	1.5	2.1	0.9	1.2	1.1	1.272
**SVM only**									
Acc %	97.00	96.20	96.20	97.00	94.20	92.40	89.87	92.33	94.40
Time (s)	1.3	4.5	1.7	4.7	5.1	5.7	11.3	8.9	5.40

**Table 2:** ANOVA of the accuracies for k-fold results.

Paper type	Beverage type classification				Beverage age classification			
	SVM only		SVM with encoder		SVM Only		SVM with encoder	
	F	p value	F	p value	F	p value	F	p value
2	0.06	0.99	0.02	0.99	0.02	0.99	0.3	0.87
3	0.04	0.99	0.004	0.99	0.02	0.99	0.96	0.43
4	0.03	0.99	0.34	0.84	0.01	0.99	0.18	0.94

**Figure 9:**
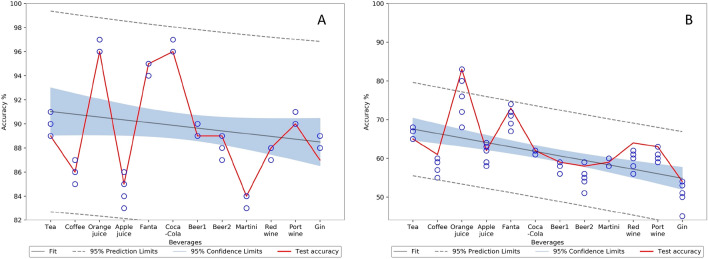
Paper3 confidence interval (CI) plot for beverage aging classification, (**A**) represents CI for SVM only and (**B**) represents CI for SVM with encoder.

The reflectance data of the same beverage stain from each time interval was used as the X variable to identify the spectra based on age, and the corresponding time interval was used as the Y variable to train the SVM model. The model was initially trained and tested using full spectrum data and obtained a very good average training accuracy of 88.75% and 88.61% on test data, the detailed accuracy obtained are given in Table 3. The SVM was then retrained using the encoder output (six dimensions) to verify the influence of dimensionality reduction and obtained a comparable average training accuracy of 61.16% and test accuracy of 59.6% with an 8 times reduction in the training time. In further analysis we find that the variations in the last three timings (1, 2 and 3 days) seems marginal for the last four drinks (port wine, martini, coffee and beer), and the SVM and CAE were misleading during training. Thus, retrained the SVM alone and then CAE with SVM by using the first six timings for all beverage stains, by eliminating the last two readings (2 days and 3 days). Which improved the SVM classification accuracy from 88.75 to 93.52% and the accuracy of the combination using encoder with SVM from 61.16 to 80.46%. The Fig. 9 depicts the CI of the paper3 aging classification and the CI of other papers are given in Appendix as Figs. 14 and 15. The Table 2 gives the ANOVA of the classification accuracies obtained over k-fold validation, all the p values are greater than 0.05 implies that there is no significant differences between the accuracies.

**Table 3:** Accuracies (average) for detecting aging of beverage stains and training time for training data set.

Beverage	SVM only		SVM only without last two timings		SVM with encoder		SVM with encoder without last two timings	
	Acc (%)	Time (s)	Acc (%)	Time (s)	Acc (%)	Time (s)	Acc (%)	Time (s)
Tea	87.27	61.4	94.73	4.1	60.53	4.2	83.00	4.1
Coffee	85.60	51.4	94.67	4.4	61.20	7.3	80.67	5.7
Orange juice	93.33	13.8	94.67	3.7	73.33	6.1	84.93	9.1
Apple juice	84.93	121.5	90.87	11.1	56.27	13.5	78.13	3.9
Fanta	95.20	34.6	95.00	15.3	77.13	10.8	84.00	11.3
Coca-Cola	94.47	25.1	95.67	14.3	66.00	9.8	82.47	8.4
Beer1	87.27	188.7	95.20	8.7	58.67	12.5	82.60	4.5
Beer2	86.00	44.3	93.60	3.4	60.40	16.4	81.67	12.8
Martini	84.67	104.84	90.40	9.1	60.07	17.9	77.80	5.6
Red wine	88.73	30.1	92.47	7.7	52.47	8.5	78.13	4.2
Port wine	88.93	25.1	93.20	4.2	56.73	9.3	80.27	3.4
Gin	88.60	175.1	91.80	20.4	51.07	13.9	71.80	7.3
Average	88.75	73.00	93.52	8.87	61.16	10.85	80.46	6.69

### Spectral changes of the substrate

**Figure 10:**
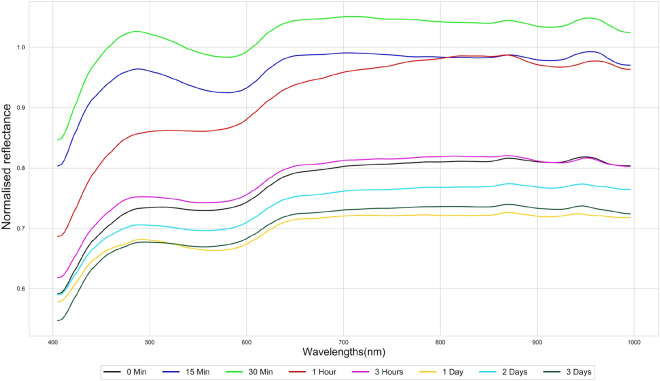
Mean spectral variation of the paper towel (paper1) over time.

**Figure 11:**
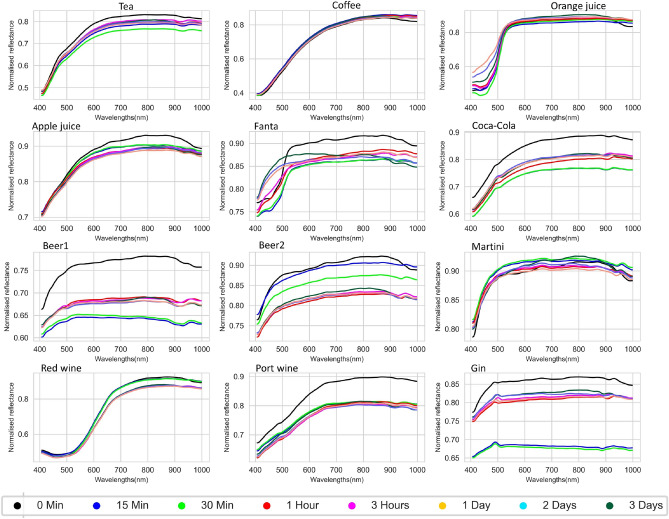
Spectra of 12 beverage stains over time (paper3).

**Figure 12:**
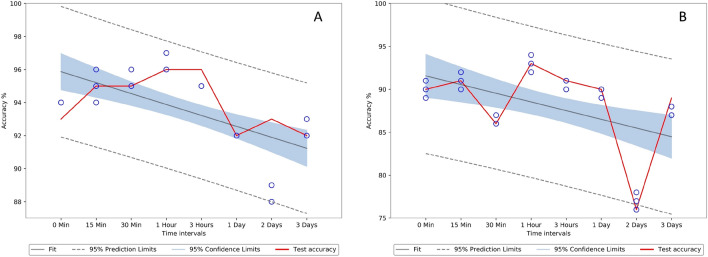
Paper2 confidence interval (CI) plot for beverage classification, (**A**) represents CI for SVM only and (**B**) represents CI for SVM with encoder.

**Figure 13:**
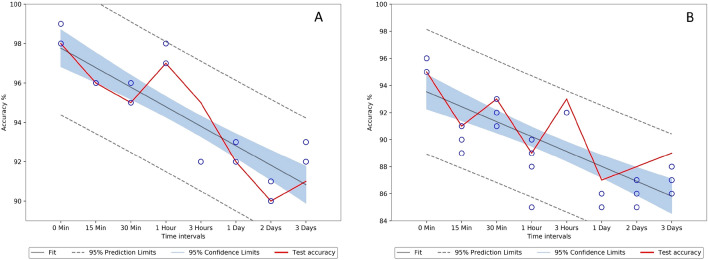
Paper4 confidence interval (CI) plot for beverage classification, (**A**) represents CI for SVM only and (**B**) represents CI for SVM with encoder.

**Figure 14:**
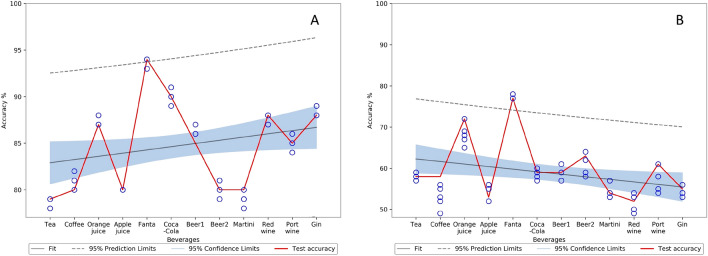
Paper2 confidence interval (CI) plot for beverage aging classification, (**A**) represents CI for SVM only and (**B**) represents CI for SVM with encoder.

**Figure 15:**
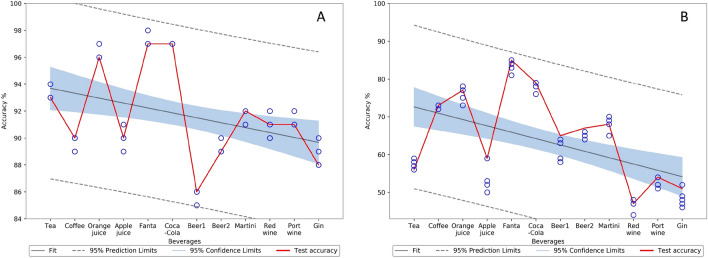
Paper4 confidence interval (CI) plot for beverage aging classification, (**A**) represents CI for SVM only and (**B**) represents CI for SVM with encoder.

We have analyzed the spectral changes in the substrate material, which is obtained from the paper towel used for sample preparation. Like the beverage samples, we selected a ROI of side 100 pixels in the paper towel and calculated the mean spectral variation which is plotted in Fig. 10. The spectrum follows a similarly identical spectral shape at different time durations, but the magnitude of the reflectance values differs over time. Which is primarily caused by the specular reflection that occurs over time from the drying of the stains. Also noticed that the spectrum has a local minima around 580 nm and a gradual rise in reflectance until approximately 630 nm and follows a nearly same reflectance value throughout the NIR range. The analysis of background spectra helps us to identify and track the beverage stains more accurately by avoiding the background spectra at each time duration.

### Optimal spectral band selection

To remove redundant details for the realization of HSI in potential real time examinations, a number of optimal wavelengths were selected using the VGBS algorithm, to create a multispectral imaging system. The data set has 186 spectral bands between 400 and 1000 nm, using VGBS we extracted a total of five wavelengths, which are 450.05, 472.3, 507.5, 871.35 and 976.6 nm. The first three bands extracted by the VGBS algorithm belong to the visible region and the last two belong to the end of the NIR region, which are aligned with our visual observation from the mean spectra of the stains. After finding these wavelengths, the SVM classifier was re-launched for both types of classification tasks and obtained the average test accuracies as 83.16% for stain detection and 60.95% for aging detection. This proves the potential of spectral selection, which can help to design a portable and cost-effective device for accurate onsite documentation of crime scenes. Compared to HSI systems, a multispectral system with limited number of selected spectral bands which can produce similar results as HSI will be very useful for forensic applications.

## Conclusions

Hyperspectral imaging was successfully used to detect, age estimation, and characterize the beverage stains on three different types of paper towels using twelve beverage types. Established classification methods using SVM and obtained promising classification accuracies. We have implemented and evaluated dimensionality reduction using CAE and analyzed the impact of lower spectral dimension on classification accuracy and processing time. We have also investigated the variations in spectral characteristics of the six beverages over 3 days as well as identified the relevant bands for classification and age estimation, using VGBS algorithm. It was observed that there are chances of challenges in capturing accurate spectral reflectance data in some locations due to specular reflections from liquid samples. The potential future work will be to extend this research with more beverages and other substrate samples of the sort generally found in crime scenes. The present study shows that HSI helps to document and examine beverage stains present in the crime scene and perform in-depth analysis. This tool also provides forensics experts the opportunity to scientifically document, reconstruct the incident, and understand the suspect´s actions before the incident.

## Data Availability

On fair request, the datasets produced during and/or analyzed during the current study are available from the corresponding authors.

## Appendix

See Figs. 11, 12, 13, 14 and 15.
